# Application of peripheral-blood-derived endothelial progenitor cell for treating ischemia-reperfusion injury and infarction: a preclinical study in rat models

**DOI:** 10.1186/1749-8090-8-33

**Published:** 2013-03-01

**Authors:** Zhi-Tang Chang, Lang Hong, Hong Wang, Heng-Li Lai, Lin-Feng Li, Qiu-Lin Yin

**Affiliations:** 1 Department of Cardiology, Jiangxi Provincial People’s Hospital, No. 92 Aiguo Road, Donghu District, Nanchang, Jiangxi 330006, People’s Republic of China

**Keywords:** Peripheral blood-derived endothelial progenitor cells, Ischemia-reperfusion injury, Left anterior descending artery, Myocardial infarction, Transplantation therapy

## Abstract

**Background:**

Our aim was to explore the therapeutic effects of peripheral blood-derived endothelial progenitor cells (PB-EPC) in cardiac ischemia-reperfusion infarction models in rats and in *in vitro* culture systems.

**Methods:**

Rat models of ischemia reperfusion and myocardial infarction were developed using male, Sprague–Dawley rats. Cardiomyocyte and endothelial cell cultures were also established. Therapeutic effects of PB-EPCs were examined *in vivo* and *in vitro* in both models. Rats underwent either cardiac ischemia-reperfusion (n = 40) or infarction (n = 56) surgeries and were transplanted with genetically modified EPCs. Treatment efficacy in the ischemia-reperfusion group was measured by infarct size, myocardial contraction velocity, and myeloperoxidase activity after transplantation. Cardiomyocyte survival and endothelial cell apoptosis were investigated *in vitro*. Vascular growth-associated protein expression and cardiac function were evaluated in the myocardial infarction group by western blot and echocardiography, respectively.

**Results:**

Infarct size and myeloperoxidase activity were significantly decreased in the ischemia-reperfusion group, whereas myocardial contractility was significantly increased in the EPC and Tβ4 groups compared with that in the control group. In contrast, no differences were found between EPC + shRNA Tβ4 and control groups. Rates of cardiomyocyte survival and endothelial cell apoptosis were significantly higher and lower, respectively, in the EPC and Tβ4 groups than in the control group, whereas no differences were found between the EPC + shRNA Tβ4 and control group. Four weeks after myocardial infarction, cardiac function was significantly better in the EPC group than in the control group. Expressions of PDGF, VEGF, and Flk-1 were significantly higher in EPC group than in control group.

**Conclusions:**

Study findings suggest that PB-EPCs are able to protect cardiomyocytes from ischemia-reperfusion or infarction-induced damage via a Tβ4-mediated mechanism. EPCs may also provide protection through increased expression of proteins involved in mediating vascular growth. Autologous peripheral-blood-derived EPCs are readily available for efficient therapeutic use without the concerns of graft rejection.

## Background

Cardiovascular disease is one of the leading causes of death in developed and developing countries. In 2008, there were 17 million deaths due to cardiovascular disease worldwide (including 7.3 million due to coronary heart disease), accounting for 30% of all global deaths [1]. Revascularization is the goal of current clinical therapy after myocardial infarction. Clinically successful revascularization urgently increases blood flow to the ischemic area to salvage cells while standard therapy is being established. Reperfusion is necessary for tissue survival, but may itself result in tissue damage. The pathophysiological process of ischemia-reperfusion injury in prolonged myocardial ischemia results in cardiomyocyte loss, microcirculatory disturbances and post-ischemic inflammation [2]. Although cardiomyocyte regeneration takes place throughout human life at about 1% per year [3], cardiomyocytes only have a limited capacity to regenerate. Most types of heart disease, especially prolonged ischemia and heart failure, are characterized by a loss of cardiomyocytes. Notably, after myocardial infarction, cardiomyocytes are incapable of sufficient regeneration to correct losses. This can lead to the development of chronic heart failure among survivors of infarction. It follows then, that therapeutic strategies that stimulate cardiomyocyte renewal are a logical target for treating cardiac pathologies [3].

Transplantation of various types of cells have been investigated as possible treatment for cardiac dysfunction after myocardial infarction [4-6], including human umbilical vein endothelial cells [4], microspheres and fibroblasts [5], and progenitor cell populations [6]. Circulating endothelial progenitor cells (EPCs) are derived from peripheral mononuclear cells and appear to play an important cardioprotective role. These cells have proliferative potential and can differentiate into mature endothelial cells. Under normal circumstances, EPCs account for approximately 0.1% of peripheral blood cells. As such, EPCs constitute a circulating pool of cells that counteract ongoing risk factor-induced endothelial cell injury and replace dysfunctional endothelium [7]. When required for vascular repair/angiogenesis or in cases of vascular stress, EPCs enter the peripheral blood and migrate to areas of endothelial damage to begin the reparative process [8]. However, after acute coronary occlusion, the supply of circulating EPCs is insufficient for neovascularization of ischemic myocardial tissues and vascular repair.

An accumulation of cardiovascular risk factors and the presence of coronary or peripheral atherosclerosis have been found to be associated with dysfunction and reduced numbers of EPCs [9-11]. Moreover, a low EPC concentration has been shown to be an independent risk factor of future cardiovascular events [7,12]. Notably, recent evidence suggests that EPCs may provide cardioprotection against acute ischemia-reperfusion injury [7,9-12].

Thymosin β4 (Tβ4) contains 43 amino acids and is abundantly expressed in EPCs [13]. Tβ4 promotes endothelial cell differentiation, tubule formation, migration, angiogenesis *in vitro*, and mediates adult cardiac repair [14]. Of particular note, the findings from a study incorporating *in vivo* and *in vitro* animal experiments suggest that the short-term cardioprotective effects of regional application of EPCs may be partially attributable to Tβ4 [15]. We aimed to confirm the previously reported findings regarding the importance of Tβ4 in mediating the effects of EPCs. Our hypothesis was that we could use EPCs isolated from peripheral blood to treat cardiomyocyte infarction. The benefits for using these cells are that they are readily available for use in treatment after purification and amplification, and that concerns about graft rejection are eliminated since the process is autotransplantation. Therefore, the purpose of this study was to explore the therapeutic effects of peripheral blood-derived endothelial progenitor cells (PB-EPCs) in rat models of cardiac ischemia-reperfusion and infarction and in *in vitro* culture systems and to further investigate the mechanisms underlying the cardioprotective effects of EPCs.

## Methods

### Animals

Sprague Dawley rats were purchased from Tongji Medical College (Wuhan, China). A total of 40 male rats were included in the ischemia-reperfusion model; 56 male rats were included in the myocardial infarction model; 16 male rats were used to establish cardiomyocyte cultures; and 14 neonatal rats were used to establish endothelial cell cultures. This study was performed in accordance with the instructions outlined in the Guidelines for the Care and Use of Laboratory Animals (US National Institutes of Health) and was approved by the Ethics Committee of Tongji Medical College, Huazhong University of Science and Technology.

### Isolation and identification of EPCs

EPCs were isolated and identified as previously described [16]. Briefly, peripheral blood obtained from rats was subjected to density gradient centrifugation to isolate EPCs. The EPCs were further processed by flow cytometry for identification and purification. Please see Zhao et al. for a detailed description of the methods [16].

### RNA interference

Tβ4 short hairpin RNA (shRNA) and scrambled (SC) shRNA were prepared using a commercially available kit (pSuperior.retro, Oligoengine Seattle, WA) as previously described [15] and EPCs were transfected with using Lipofectamine 2000 reagent (Life Technologies, Carlsbad, CA). Transfection efficiency was confirmed by real-time polymerase chain reaction as previously described [15].

### Rat ischemia-reperfusion model

Ischemia-reperfusion was induced in 40 male Sprague–Dawley rats (weight: 230 to 350 g; aged: 7 to10 weeks) via the following procedure. Rats were anesthetized with ketamine (100 mg/kg) and xylazine (10 mg/kg). The chest was opened by middle thoracotomy. After pericardiotomy, a 4–0 black silk ligature was placed under the left anterior descending (LAD) coronary artery, and the ends of the tie were threaded through a small vinyl tube to form a snare to facilitate reversible LAD coronary artery occlusion for 40 min. Myocardial ischemia was confirmed by the appearance of regional epicardial cyanosis over the myocardial surface and by arrhythmia. After 35 min of ischemia, rats received intramyocardial injection into the infarct and peri-infarct regions of the heart. Reperfusion was established by loosening the snare. Successful reperfusion was confirmed by the disappearance of epicardial cyanosis, epicardial hyperemia, and arrhythmia [17]. Muscle and skin incisions were closed using 3–0 silk absorbable suture. Rats received postoperative buprenorphine and cefazolin (0.1 mg/kg and 40 mg/kg, respectively) via subcutaneous injection for 5 days. Operation site spray and topical antibiotic powder was applied to the wound to prevent infection. Intraperitoneal injections of the immunosuppressive agent cyclosporine were given to all rats daily for 4 days before cell transplantation and for 6 weeks after transplantation [18]. Infarct size, MPO assay and assessment of hemodynamics and myocardial contractility were assessed as described below.

#### Myeloperoxidase assay

At 24 hours after reperfusion, rats were anesthetized as described above. The chest was then opened at the fourth intercostal space and the heart was removed by applying gentle pressure to either side of the incision. The LAD coronary artery was permanently occluded and 2 mL of Evans blue (1% in saline) was infused via the jugular vein to determine the area at risk (AAR), non-ischemic area and infarct size. The AAR, non-ischemic area and infarct region were homogenized in buffer containing 20 mM sodium phosphate buffer (pH 4.7), 0.015 M EDTA, and 0.1M sodium chloride before centrifugation at 10,000 rpm for 15 minutes at 4°C. MPO activity was defined as the quantity of enzyme degrading 1 μmol of peroxide per minute at 25°C and is expressed as units per gram of weight [19].

#### Hemodynamics and myocardial contractility

At 72 hours after reperfusion, rats were anesthetized as described above and the right common carotid artery was cannulated with a 2-Fr Millar catheter. The catheter was advanced into the left ventricle (LV) to measure the heart rate (HR), left ventricular systolic pressure (LVSP), left ventricular end diastolic pressure (LVEDP), the increase in the peak rate of intraventricular pressure increase (+dP/dtmax), and the decrease in the peak rate of intraventricular pressure (−dP/dtmax) [20].

### Rat myocardial infarction model

Myocardial infarction was induced in 56 male rats (weight: 230 to 350 g; age: 7 to 10 weeks) randomly divided into an EPC treatment group (n = 28; 150 μL containing 5 × 10^6^ cells) and a control group (n = 28). Rats were anesthetized by intraperitoneal injection of ketamine (70 to 80 mg/kg). The analgesic buprenorphine was given preoperatively (10 to 20 μg/kg body weight, intraperitoneal). The rats were connected to a respirator via tracheotomy. The right external jugular vein was cannulated for systemic intravenous administration of treatment. A left-sided thoracotomy was performed by cutting the fifth and sixth ribs. The pericardium was opened, and the heart exteriorized with a cardiac holder consisting of a plastic loop (1.5 × 2 cm). The LAD was localized 1–2 mm below the junction of the pulmonary conus and the left atrial appendage [21], and was ligated from the left border of the pulmonary conus to the right border of the left atrial appendage using 5–0 silk suture. The heart was moved from its exteriorized position to its normal position in the chest cavity. Presutured loops of 4–0 silk were used to close the chest wall [21]. Postoperative care and immunosuppressive treatment was done as per the ischemia-reperfusion model except that immunosuppressive treatment was continued for up to 4 weeks as necessary. Cardiac function was assessed as shown below.

#### Cardiac function

Echocardiograms to assess systolic function were performed using M-mode and two-dimensional measurements. Rats were anesthetized with 5% isoflurane (3.5 L/m O_2_ for 30 s) followed by 2% isoflurane and O_2_ for an average of 10 to 15 min. Echocardiographic measurements were made 4 to 7 mins after the induction of anesthesia to allow for resolution of any transient anesthesia-related cardiac depression. Measurements comprised the average of six selected cardiac cycles from at least two separate scans performed in a random-blind fashion with papillary muscles used as a reference point for consistency in scan levels. End diastole was defined as the maximal left ventricle diastolic dimension and end systole was defined as the peak posterior wall motion. Single outliers in each group were omitted for statistical analysis. Fractional shortening, (FS%), a surrogate of systolic function, was calculated from LV dimensions as follows: FS% = (LVED-LVES)/LVED × 100. LVED and LVES are LV dimensions at end diastole and end systole, respectively. The ejection fraction (EF) was calculated from two-dimensional images.

#### Determination of area at risk and infarct size

After analyzing hemodynamic function, the LAD coronary artery was relegated to its original position and treated as described above to identify myocardial AAR. The atria, right ventricles, and major vessels were removed. The LV was then sliced transversely into 2 mm thick slices. To distinguish between ischemic and non-ischemic tissue, the slices were incubated in 1% triphenyltetrazolium chloride at 37°C, pH 7.4 for 20 min. AAR pieces were separated according to the extent of staining and weighed to determine the infarct size as a percentage of the weight of the AAR. AAR was expressed as a percentage of the LV as previously described [17].

#### Western-blot analysis

Sternotomy was performed on rats (n = 6 per group) 24 hours after myocardial infarction. Heart tissue was harvested from non-infarct regions of the heart. Denatured cell lysates (50 μg) were resolved on a 10% sodium dodecylsulfate polyacrylamide gel and then transferred to nitrocellulose membranes. The membranes were blocked with 5% non-fat dry milk in Tris buffered saline for 2 hours at room temperature. Thereafter, membranes were incubated with primary antibodies (1:2000) against fibroblast growth factor-17 (FGF-17), fibroblast growth factor receptor-2 (FGFR-2), β-Catenin, vascular endothelial growth factor (VEGF), fetal liver kinase-1 (Flk-1), Tbx-18, and platelet-derived growth factor (PDGF) (Santa Cruz Biotechnology, Santa Cruz, CA) at 4°C overnight. After being washed three times for 10 min each in TBS–0.05% Tween 20, the membranes were incubated with horseradish peroxidase conjugated secondary antibody (1:3000) for 2 hours at room temperature. Specific bands were detected using the Pierce ECL detection system (Thermo-Fischer Scientific, Beijing, China). Band intensities were quantified with NIH Image Version 1.61. GAPDH was used as an internal control.

### Cardiomyocyte and endothelial cell cultures

For culture of cardiomyocytes, 16 rats were sacrificed (intraperitoneal sodium pentobarbital: 120 mg/kg) for endothelial cell culture and hearts were quickly excised via midline thoracotomy. Ventricular cardiac muscle cells were isolated and plated in medium 199 with 4% fetal calf serum on glass coverslips as previously described [13]. Two to three coverslips were used for each isolation. Approximately 4 hours after plating, the cells were washed with medium 199 and then exposed to 4 hours of hypoxia and then 1 hour of reoxygenation. The EPCs were placed in a transwell/permeable insert in the well before hypoxia. At the end of the experiment, Trypan blue was added for 2 minutes, and 5 microscopic fields were photographed to quantify cell survival.

For culture of endothelial cells, 14 2- to 3-day-old neonatal rats (preferred for superior viability of isolated cells) were sacrificed by cervical dislocation under anesthesia using pentobarbital (40 mg/kg, intraperitoneal). Coronary endothelial cells were harvested from rat hearts by collagenase and trypsin digestion. After seeding for 1 hour on collagen-coated dishes, nonadherent cells were removed and incubated with DMEM with 10% PBS as previously described [15]. EPCs, with or without Tβ4 shRNA transfection, were added to the cultures. Coronary endothelial cells were exposed to 18 hours of hypoxia, followed by 4 hours of reoxygenation and then fixed with 4% paraformaldehyde. The extent of apoptosis was determined using a commercial terminal deoxynucleotidyl transferase–mediated dUTP nick-end labeling staining kit.

### Statistical analysis

Data are presented as mean with standard deviation. Comparisons were performed using ANOVA with post-hoc comparison adjusted using the Bonferroni method. Data were analyzed using SPSS 15.0 statistics software (SPSS Inc, Chicago, IL). *P* values < 0.05 were considered to be statistically significant.

## Results

### Ischemia-reperfusion model

#### Infarct size and AAR

**Figure 1:**
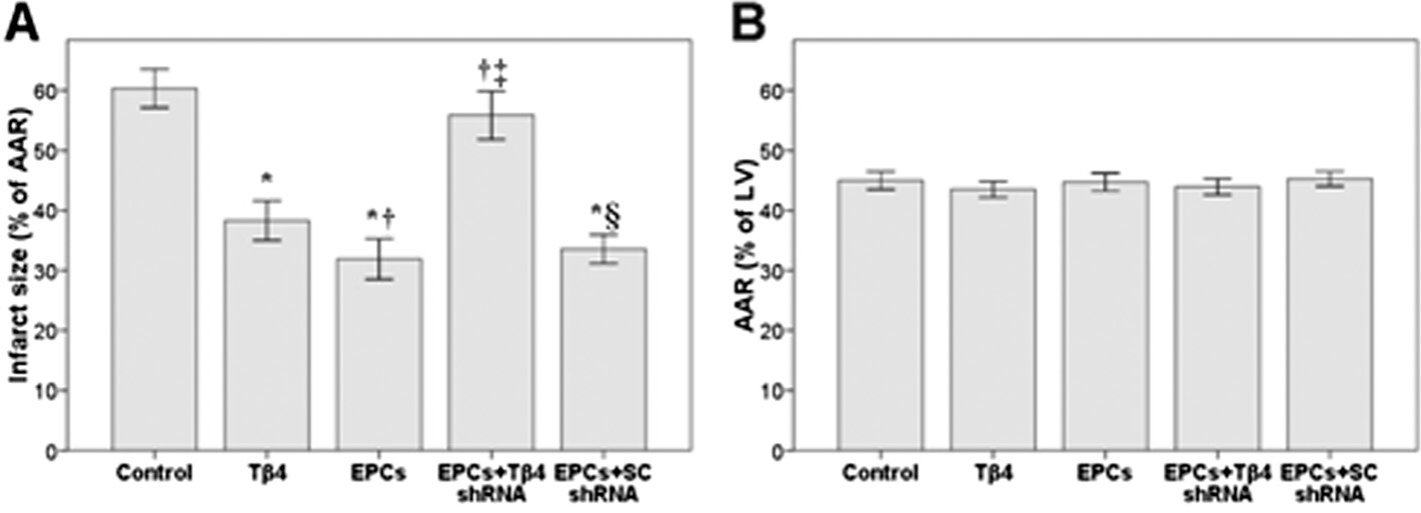
**The effect of EPC and Tβ4 in a myocardiac infarction model.** Infarct size (**A**) and area at risk (AAR: **B**) in rats 72 hours after ligating the LAD coronary artery. Rats were treated with: endothelial progenitor cells (EPCs) (150 μL, 5 × 10^6^ cells per rat); EPCs transfected with Tβ4 short hairpin RNA (shRNA) (150 μL, 5 × 10^6^ cells per rat); EPCs transfected with scrambled (SC) shRNA (150 μL, 5 × 10^6^ cells per rat); or 6 mg Tβ4. Control rats were untreated. *P < 0.05 compared with the control group; †*P* < 0.05 compared with the Tβ4 group; ‡*P* < 0.05 compared with the EPC group; § *P* < 0.05 compared with the EPC + Tβ4 shRNA group. Data are presented as mean ± standard deviation (n = 8 per group).

The impact of Tβ4/EPCs on infarct size and AAR is summarized in Figure 1. Three days after injection, infarct size was significantly reduced in the Tβ4 (38 ± 3%) and EPC (32 ± 3%) groups compared with that in the control group (60 ± 3%; Figure 1A). Intramyocardial injection of EPCs with SC shRNA resulted in similar cardioprotective effects to EPCs-alone group. Knockdown of Tβ4 mRNA by Tβ4 shRNA reversed the EPC-induced decrease in infarct size compared with that in the EPCs group (*P* < 0.05). No significant differences were found in infarct size between the EPC group and the EPCs + SC shRNA group. The extension of the infarct at risk (AAR/left ventricle [LV] area) did not differ significantly between groups (Figure 1B).

#### Hemodynamics and myocardial contractility

**Figure 2:**
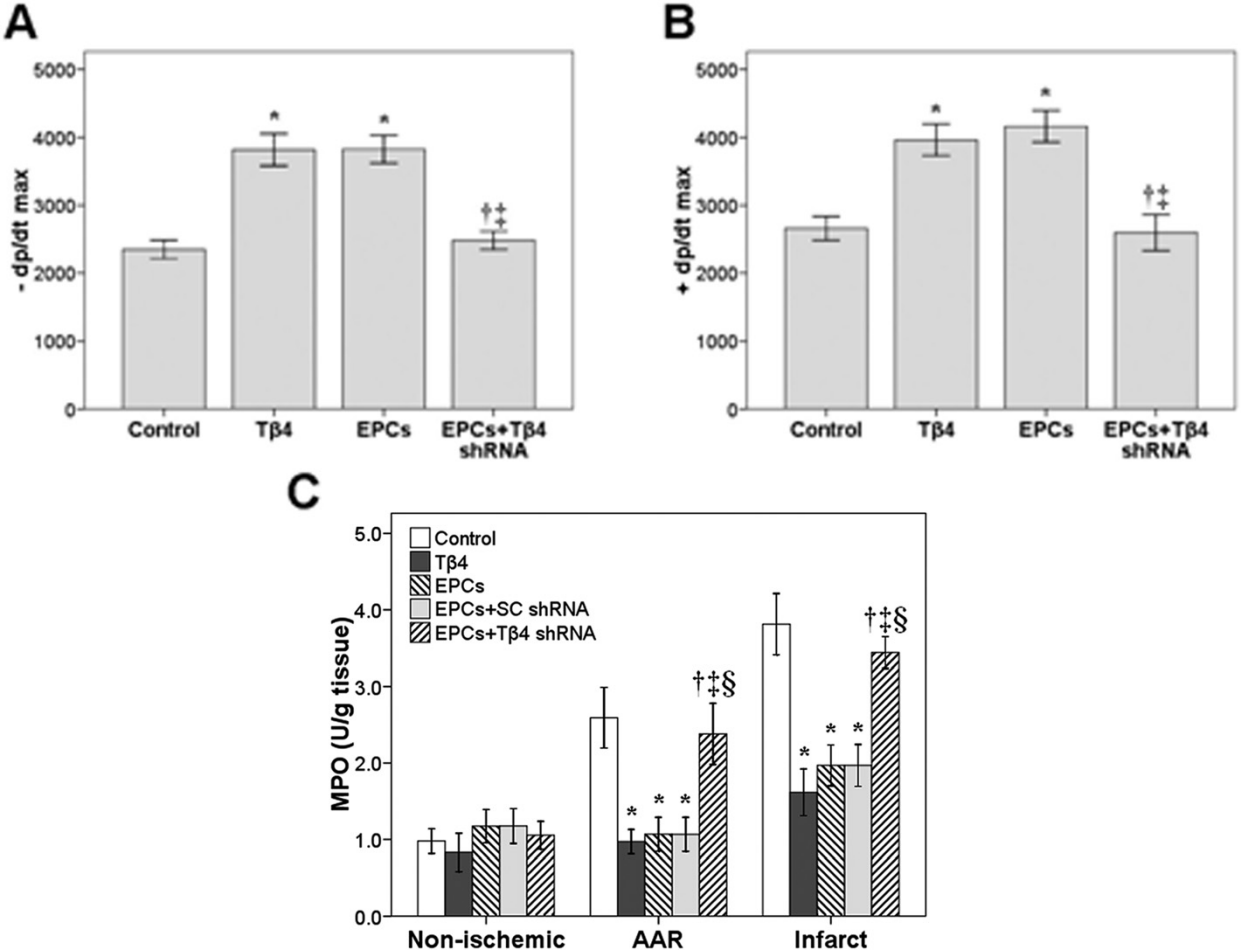
**Measurement of cardiac functions and myeloperoxidase activities.** Increase in the peak rate of intraventricular pressure increase (+dP/dtmax: **A**), decrease in the peak rate of intraventricular pressure (−dP/dtmax: **B**) in rats 72 hours, and cardiac myeloperoxidase activity (MPO: **C**) in rats 24 hours after ischemia-reperfusion injury. Rats were treated with: endothelial progenitor cells (EPCs) (150 μL, 5 × 10^6^ cells per rat); EPCs transfected with Tβ4 short hairpin RNA (shRNA) (150 μL, 5 × 10^6^ cells per rat); EPCs transfected with scrambled (SC) shRNA (150 μL, 5 × 10^6^ cells per rat); or 6 mg Tβ4. Control rats were untreated. *P < 0.05 compared with the control group; †*P* < 0.05 compared with the Tβ4 group; ‡*P* < 0.05 compared with the EPC group; §*P* < 0.05 compared with the EPCs + SC shRNA group. Data are presented as mean ± standard deviation (n = 8 per group).

The impact of Tβ4/EPCs on myocardial contractility is summarized in Figures 2A and 2B. Both -dP/dtmax and + dP/dtmax were significantly increased in the EPCs and Tβ4 groups compared with those in the control group (*P* < 0.05). Conversely, Tβ4 shRNA inhibited the effect of EPCs for both variables (EPC vs. EPCs + Tβ4 shRNA, *P* < 0.05).

#### Myeloperoxidase activity

The impact of Tβ4/EPCs on MPO activity in non-ischemic, AAR, and infarct tissue is summarized in Figure 2C. MPO activity in AAR and infarct tissue was significantly reduced in the Tβ4 group compared with that in the control group (*P* < 0.05). Similar reductions were seen in the EPCs + SC shRNA group (*P* < 0.05) and the EPC group (*P* < 0.05). Notably, Tβ4 shRNA blocked the inhibitory effect of EPCs on MPO activity (EPCs + Tβ4 shRNA vs. EPCs + SC shRNA, *P* < 0.05).

### Cardiomyocyte and endothelial cell culture

**Figure 3:**
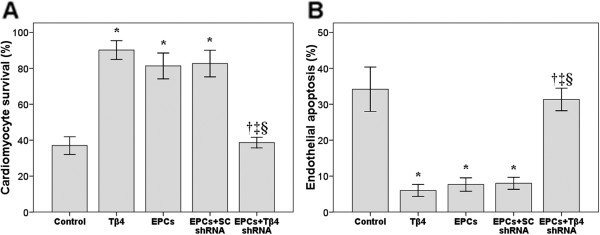
**Measuring EPC function in an *in vitro *assay.** Cardiomyocyte survival (**A**) and endothelial cell apoptosis (**B**) after exposure to hypoxia-reoxygenation (18 h-4 h) ischemia-reperfusion. Cells were incubated with: endothelial progenitor cells (EPCs); EPCs transfected with Tβ4 short hairpin RNA (shRNA); EPCs transfected with scrambled (SC) shRNA in transwell plate; Tβ4 were added directly into culturing well. Control cells were not treated beside hypoxia-reoxygenation *P < 0.05 compared with the control group; †*P* < 0.05 compared with the Tβ4 group; ‡*P* < 0.05 compared with the EPC group; §*P* < 0.05 compared with the EPCs + SC shRNA group. Data are presented as mean ± standard deviation (n = 6 per group).

The impact of Tβ4/EPCs on cardiomyocyte survival and endothelial cell apoptosis after hypoxia-reoxygenation is summarized in Figure 3. Cardiomyocyte survival was significantly increased in the Tβ4 and EPCs + SC shRNA groups compared with that in the control group (both *P* < 0.05; Figure 3A). Tβ4 shRNA blocked the protective effect of EPCs on cardiomyocyte survival (EPCs + Tβ4 shRNA vs EPCs + SC shRNA, *P* < 0.05). Endothelial cell apoptosis was significantly decreased in the Tβ4 and EPCs + SC shRNA groups compared with that in the control group (both *P* < 0.05; Figure 3B). Tβ4 shRNA blocked the protective effect of EPCs on endothelial cell apoptosis (EPCs + Tβ4 shRNA vs EPCs + SC shRNA, *P* < 0.05).

### Myocardial infarction model

#### Cardiac function

The impact of EPCs on cardiac function at 2 and 4 weeks after myocardial infarction is summarized in Table 1. At both 2 and 4 weeks after infarction, the end diastolic and end systolic dimensions were significantly lower in the EPC group compared with those dimensions in the control group (*P* < 0.05). In contrast, fractional shortening and ejection fraction were significantly higher in the EPC group compared with those in the control group at the given weeks (*P* < 0.05).

**Table 1:** Measures of cardiac function in rats with myocardial infarction who received treatment with endothelial progenitor cells

**Time**	**Groups**	**EDD**	**ESD**	**FS**	**EF**
Week 2	Control (n = 28)	7.01 ± 0.27	4.11 ± 0.17	46.41 ± 2.25	31.67 ± 2.94
	EPCs (n = 28)	6.01 ± 0.19*	3.09 ± 0.18*	60.51 ± 2.17*	66.18 ± 2.16*
Week 4	Control (n = 28)	6.57 ± 0.28	3.66 ± 0.34	43.49 ± 3.07	35.37 ± 2.39
	EPCs (n = 28)	5.69 ± 0.24*	2.64 ± 0.33*	56.48 ± 2.68*	68.39 ± 3.89*

*EPC*, endothelial progenitor cell; *EDD*, end diastolic dimension; *ESD*, end systolic dimension; *FS*, fractional shortening; *EF*, ejection fraction.

* Indicates a significant difference between the EPC and control group (*P* < 0.05).

#### Western blot analysis

**Figure 4:**
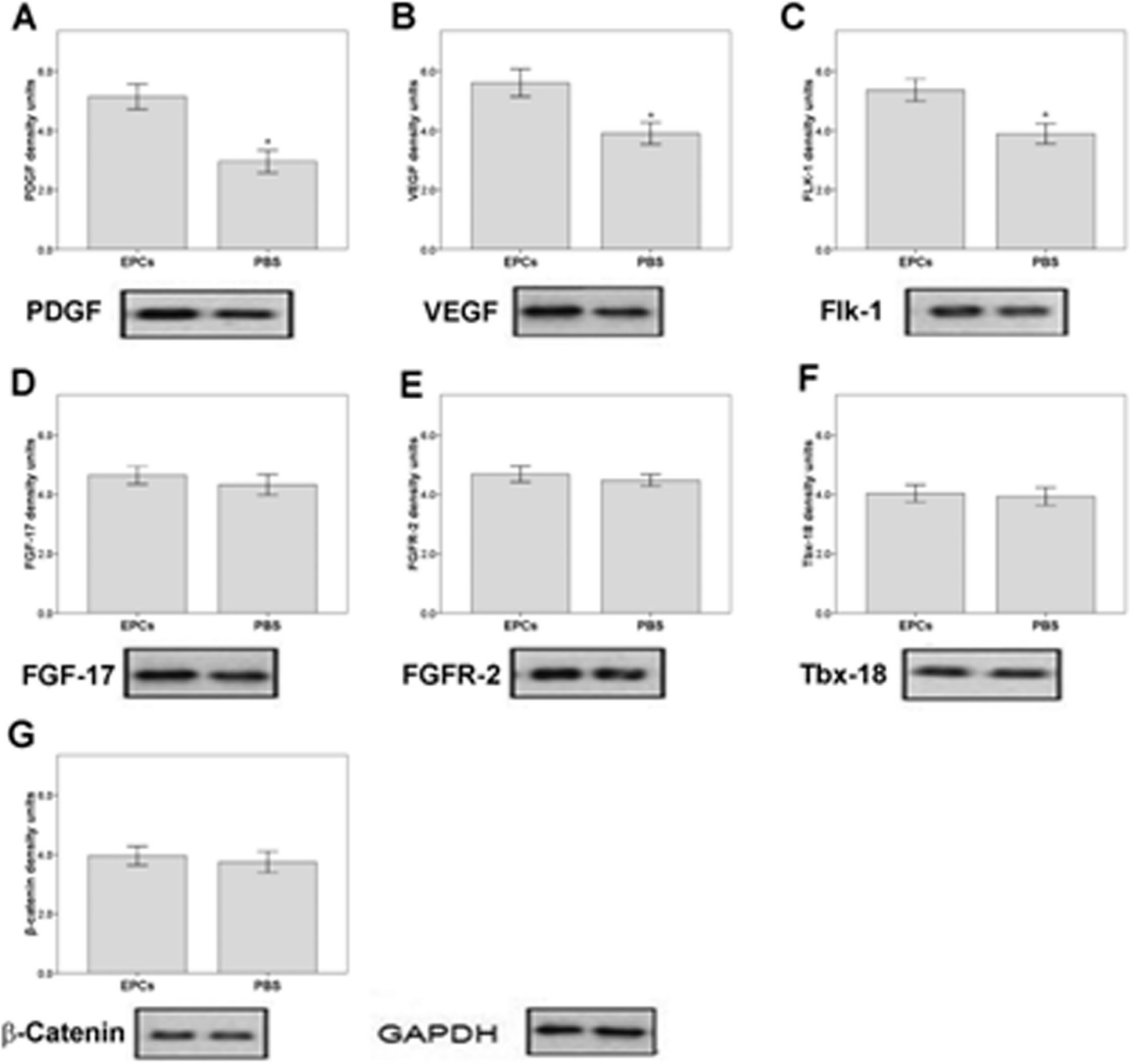
**EPC treatment induced cardioprotection related proteins expression.** Protein expression of platelet-derived growth factor (PDGF: **A**), vascular endothelial growth factor (VEGF: **B**), fetal liver kinase-1 (Flk-1: **C**), fibroblast growth factor-17 (FGF-17: **D**), fibroblast growth factor receptor-2 (FGFR-2: **E**), Tbx-18 (**F**), and β-Catenin (**G**) in heart tissue from rats 24 h after myocardial infarction. Rats were treated with endothelial progenitor cells (EPCs) or were untreated (controls). Protein expression levels were determined by Western blot using heart tissue harvested from non-infarct areas. Density units were normalized to the GAPDH loading control. *P < 0.05 compared with the EPC group. Data are presented as mean ± standard deviation (n = 6 per group).

The impact of EPCs on the expression of vascular growth-associated proteins in heart tissue 24 hours after myocardial infarction is summarized in Figure 4. Expressions of PDGF, VEGF, and Flk-1 were significantly higher in the EPC group compared with those in the control group (all *P* < 0.05). No other significant between-group differences were found in protein expression levels.

## Discussion

To investigate the effects of treatment with peripherally-derived EPCs on myocardial infarction *in vitro*, we evaluated whether PB-EPCs treatment might increase myocardial repair after acute coronary occlusion in two rat models. PB-EPCs were shown to reduce infarct size, decrease ischemia/reperfusion injury, attenuate inflammation, and improve post-infarction cardiac function.

### PB-EPCs reduced infarct size and decreased ischemia-reperfusion injury

In the present study, Tβ4-transfected PB-EPCs reduced infarct size and increased regional myocardial contraction reserve in an LAD occlusion model. The impact of Tβ4 or EPCs on infarct size was assessed as the ratio of nonviable myocardium to the AAR, revealing that transplantation of PB-EPCs decreased ischemia/reperfusion injury in a rat model. The ability of Tβ4 or EPCs to prevent cell death within 72 hours after coronary ligation likely leads to the decreased infarct size and explains the improved regional myocardial contraction reserve observed in rats in this study. Incubation of PB-EPCs with Tβ4 resulted in an increase in cell viability and proliferation and had an inhibitory effect on EPCs apoptosis. Our findings are consistent with those of Bock-Marquette et al. (2004) in which Tβ4 stimulated migration of cardiomyocytes and endothelial cells, showing enhanced survival of embryonic and postnatal cardiomyocytes cultured with Tβ4 [22]. Investigators explained that the LIM proteins ILK and PINCH, which are both essential in cell migration and survival, form a complex with Tβ4 that results in phosphorylation of Akt; in mice, this activity enhanced early myocyte survival and improved cardiac function, which may point to activation of Akt as the dominant mechanism by which Tβ4 promotes cell survival [22]. Activation of ILK and subsequent stimulation of Akt may, in part, explain the enhanced cardiomyocyte survival induced by Tβ4 in the present study, although further experiments are required to confirm this. Besides the ability of Tβ4 to mediate angiogenesis in a long-term course [23], Tβ4 is integrally involved in cell motility, migration and survival in the process of cardiac morphogenesis. Results of our experiments and others indicate that this protein can be reintroduced in order to reduce post-infarction loss of cardiomyocytes and may ultimately reduce the impact of prolonged ischemic injury. However, because we did not use Tβ4 and its blocker in chronically infarcted rats, we cannot conclude that Tβ4 is involved in the chronic phase of myocardial infarction.

### PB-EPCs promote expression of proteins involved in cardiac repair and regeneration

In this study, we observed that PB-EPCs increased or activated the expression of proteins essential for cardiac repair and regeneration. Considering the multifaceted roles of VEGF, PDGF, β-Catenin, FGFR-2, FGF-17 and Flk-1 in vessel formation [24-27], our findings suggest that EPCs may promote cardiac regeneration after cardiac injury, especially given that EPC transplantation significantly increased the expression level of VEGF in the infarct region. We detected significant changes in PDGF, VEGF, and Flk-1 in the group receiving EPCs alone. Upregulated expression of VEGF has previously been found to be associated with a significant increase in the number of both capillaries and arterioles after cardiac injury [28,29]. Therefore, our finding is consistent with that previously reported in a swine model, which showed that EPCs increased neovascularization in the infarct region border zone [30]. Those investigators described a novel mechanism of cytoprotective gene expression that can potentially extend cell survival at the genomic level, and further suggested that a repetitive pattern of ischemia-reperfusion may result in progressive cell protection and survival. These results and ours suggest that activation and increased expression of individual genes plays an important role in cardioprotection.

### PB-EPCs attenuate inflammation

In this study, we observed a decrease in post-ischemic inflammation and endothelial apoptosis and significantly enhanced cardiomyocyte survival after intramyocardial injection of PB-EPCs transfected with scrambled SC shRNA and of Tβ4 alone. Tβ4 alone significantly reduced MPO activity, and similar inhibition in MPO activity was observed in the EPCs + SC shRNA group, resembling the effect of EPCs alone. In another study, Tβ4-expressing cells found in cardiac valve precursors known as “endocardial cushions” also expressed Akt as a possible interacting protein and part of the dominant mechanism by which Tβ4 increases cell survival [22]. The Tβ4 protein was also found in the ventricular septum, the compact layer of the trabecular region and the outflow tract myocardium, indicating its presence in migrating cells [22]. We believe that the effect of injected Tβ4 to stimulate migration of myocardial cells was also true in the present study.

Inflammation is now recognized as an important contributing factor to atherosclerosis and heart disease [31]. A strong positive correlation also has been observed between the progression of coronary artery disease (CAD) and circulating EPC levels, which are significantly lower in patients with angiographic CAD progression [32]. In fact, reduced levels of circulating EPCs may predict cardiovascular disease progression. Ischemia-reperfusion injury also is noted to have a marked inflammatory response, including the release of cytotoxic substances, oxygen-derived free radicals and proteases [17]. The Tβ4 protein is angiogenic, helping to form new vessels from existing vasculature, and by this mechanism has been shown to accelerate wound healing and reduce inflammation [23]. Does it also have other antiinflammatory effects? In a rat model of ischemia-reperfusion injury, daidzein, an isoflavone found in soybeans, was administered *in vivo*, attenuating myocardial damage by inhibiting nuclear factor Kappa-b (NF-kB) activation, which in turn suppressed activation of pro-inflammatory cytokines [17]. Infarct size and inflammation were reduced and cardiac function was significantly improved in the daidzen-treated rats; conversely, reperfusion after ischemia activated NF-kB and increased levels of pro-inflammatory cytokines. Another study showed that flavonoids protect against ischemia-reperfusion injury by scavenging reactive oxygen species (ROS), demonstrating protective efficacy in cardiomyocytes [33]. However, it remains to be seen whether antiinflammatory effects alone are sufficient to reduce infarct size and improve cardiac function.

### PB-EPCs improve post-infarction cardiac function

Transplantation of different cell populations, including purified cardiomyocytes [34], human induced pluripotent stem cells (iPS cells) [35], and EPCs [36] have all shown improved cardiac contractile functioning. In the present study, which used EPCs isolated from autologous peripheral blood to treat cardiomyocyte infarction, random-blind ultrasonography with multiple measurements of cardiac contraction was performed in all surviving adult rats in the myocardial infarction model. At 4 weeks post-infarction, the LV fractional shortening in the EPCs-treated group was larger than that of rats in the control group. Two-dimensional echocardiographic measurements of ventricular function also revealed that the mean ejection fraction from the left ventricle in the EPCs-treated group rats after coronary ligation was larger than that of rats in the control group. ESDs and EDDs in rats receiving EPC treatment were significantly improved, suggesting that EPCs treatment resulted in decreased cardiac dilation after myocardial infarction—a sign of improved cardiac function. Reduced remodeling of the heart after ischemic injury followed transplantation of undifferentiated human iPS cells into immunosuppressed mice; although it resulted in tumor development, the cells further differentiated into cardiac cells *in vivo*, with a trend of declining EF compared to control rats with infarction [37]. A study investigating post-infarction contractile function of the heart via 2D echocardiography, significantly impaired LV function was improved by 3% after EPCs infected with stromal-cell derived factor-1α (SDF-1α) were transplanted; SDF-1α promoted the proliferation and migration of the EPCs into injured tissue [36]. EPCs maintained their protection against progressive LV dilatation and dysfunction compared to the MI control group. The major mechanism appears to be paracrine pathways of intramyocardial injection EPCs, resulting in the prevention of apoptosis and enhanced neovascularization of ischemic myocardium [37]. Applying this understanding, we can infer that the ability of PB-EPCs to prevent cell death after coronary ligation probably leads to decreased infarct size and improved ventricular function as observed in rats in the present study.

## Conclusion

Results of this study suggest that PB-EPCs are able to protect cardiomyocytes from ischemia-reperfusion or infarction-induced damage in rat models via a Tβ4-mediated mechanism. PB-EPCs may also provide protection through increased expression of proteins involved in mediating vascular growth. Autologous peripheral-blood-derived EPCs are readily available for efficient therapeutic use without the concerns of graft rejection. Findings of this study enhance the present understanding of mechanisms underlying the cardioprotective effects of EPCs in ischemia-reperfusion injury and myocardial infarct.

## Abbreviations

PB-EPC: Peripheral blood-derived endothelial progenitor cells; EPCs: Endothelial progenitor cells; Tβ4: Thymosin β4; shRNA: Short hairpin RNA; LAD: Left anterior descending; AAR: Area at risk; LV: Left ventricle; HR: Heart rate; LVSP: Left ventricular systolic pressure; LVEDP: Left ventricular end diastolic pressure; FS%: Fractional shortening; EF: Ejection fraction; FGF-17: Fibroblast growth factor-17; FGFR-2: Fibroblast growth factor receptor-2; VEGF: Vascular endothelial growth factor; Flk-1: Fetal liver kinase-1; PDGF: Platelet-derived growth factor; CAD: Coronary artery disease; NF-kB: Nuclear factor Kappa-b; ROS: Reactive oxygen species; iPS cells: Induced pluripotent stem cells.

## Competing interests

The authors have no conflicts of interest to declare.

## Authors’ contributions

ZTC was the guarantor of integrity for the entire study and was responsible for study design, literature research, experimental studies and data analysis. LH contributed study concepts and clinical studies. HW participated in statistical analysis and manuscript preparation. HLL was responsible for manuscript editing, LFL for data acquisition and QLY for manuscript review. All authors read and approved the final manuscript.
